# The Antiseptic and Disinfectant Properties of Soap

**Published:** 1900-03

**Authors:** 


### Abstracts and Translations.

### THE ANTISEPTIC AND DISINFECTANT PROPERTIES OF SOAP.

THE *British Medico-Chirurgical Journal* for September, 1899, has in its pages an article by Symes upon this somewhat homely but practical topic.

With regard to the question, "Can germs live and multiply on soap?" he says that all soaps possess antiseptic properties in greater or less degree. The following experiments serve to illustrate this fact: (1) Fragments taken from the centre of a cake of soap by means of a sterile cork borer, and incubated in nutrient broth, were found in all cases to be sterile. (2) The hands were washed in hot tap-water with each soap; the tablet was placed on a clean surface, and cultures made from the soap at the expiration of three minutes. Those from germicidal and from scrubbing carbolic soap were sterile, and those from izal, toilet carbolic, lysol, and brown Windsor soaps showed growth of various organisms. (3) Small slabs of each soap were moistened and then heavily inoculated with a culture of staphylococcus aureus, and kept in a moist hot chamber for two days. At the expiration of this time the surfaces of the brown Windsor, lysol, and germicidal soaps were sterile, but scrapings from the others all gave rise to growth of the organism first inoculated. On none of the surfaces was there any apparent increase of growth, nor did he find it possible to grow moulds or bacteria on surfaces of soap kept under ordinary conditions. We may conclude, then, that organisms which get rubbed into a soap in the process of washing hands, clothes, or other surfaces, or which may settle upon soap from the air, are not capable of multiplication thereon. Of the soaps tested, this antiseptic property was most marked in that containing biniodide of mercury.

For practical purposes the second point—namely, the disinfectant value of soaps—is the more important. To test this the following method was adopted: A one per cent. solution of each soap was made (this representing what the writer judged to be the strength of the solution which comes into contact with the hands), and to five cubic centimetres of this solution there was added a drop of a fresh broth culture of staphylococcus. The tube was then shaken and allowed to stand for a stated period, and then five drops of the mixture were added to a broth tube, which was incubated for forty-eight hours. Obviously, if the antiseptic property of the soap solution was sufficient to kill the organisms in the one drop of broth culture added, then the tubes inoculated from the mixture should be sterile; whilst if the solution had no antiseptic power, or if the time allowed was insufficient, then growth would occur.

Symes does not give the details of many experiments extending over several months, but simply states the result arrived at, viz., that tested in this way it was found that a one per cent. solution of germicidal soap killed staphylococcus aureus in one minute, whilst the same strength of izal, toilet carbolic, scrubbing carbolic, lysol, and brown Windsor soaps failed to do so in ten minutes, half an hour, an hour, or three hours. These solutions were, however, all sterile in from twelve to fourteen hours; the exact time in which this result was attained was not observed, nor is it of much importance, for under no conditions would objects be as long as three hours in contact with the soap.

It is a matter of some importance to note that all organisms are not affected alike by soap solutions. Thus the cholera vibrio, the typhoid bacillus, the bacillus coli, and the streptococcus are killed much more quickly, or by very much more diluted solutions, than are the staphylococci. For instance, the bacillus coli is killed by a two per cent. solution of plain curd soap in from two to four hours. Our antiseptic precautions are, however, commonly directed against the more resistant organisms, the staphylococci, and therefore in testing the germicidal power of a soap it is preferable to work with these organisms. Symes has tested the germicidal soap with bacillus coli, bacillus typhosus, the cholera bacillus, streptococcus and staphylococcus albus, all of which were killed by admixture with a one per cent. solution (equal to biniodide of mercury 1 in 5000) in one minute.

It may be concluded, then, from these experiments, that for practical purposes most of the so-called disinfectant soaps have no value, but that in the combination of biniodide of mercury with soap we have a useful means of disinfecting hands, instruments, surfaces, etc.

Although a large number of trials were made, Symes did not succeed in sterilizing his hands by washing with the soap containing biniodide of mercury, although much better results were obtained with this than with any other variety. This points to the necessity of the operator first washing his hands and then soaking them in an antiseptic solution.

It has been thought that the germicidal action of soaps is due to their alkalinity, especially to the free alkali present. Symes does not think that this can be the case, for Dr. Munro, from a careful analysis of the samples tested, found that the difference in the amount of free alkali is infinitesimal. Moreover he obtained no better results with soaps with high total alkalinity than with the others.

Although the exact combinations formed are not known, there are many observations to prove that certain antiseptics when mixed with soap partly lose their power. This is certainly the case with carbolic acid, lysol, and izal. Rideal, who has done much work on this subject, considers that for an antiseptic soap an olein base is the best. Superfatted soaps are in his opinion not so suitable vehicles for antiseptics as soaps with a moderate excess of alkali. The presence of free fat or oil strongly militates against germicidal action—witness Koch's discovery that carbolized oil has no antiseptic value. Acids and free halogens are incompatible with the fat. Boracic acid is converted into sodium borate, and most mercury salts into insoluble mercuric oleate. Oleates do not generally mix well with soap; fluorides, sulphates, and oxides give better results. Rideal found the double iodide of mercury and potassium to mix well and form a good antiseptic with soap, compatible with strong alkalies and not precipitating albumin. In composition this resembles the soap with which were obtained the best results.

In conclusion, Symes points out that the matter is one of considerable importance with regard to nurses, attendants upon sick persons, and the general public, who may be led to think that in using so-called antiseptic soaps they are insuring efficient disinfection. There is also an economic side of the question, for most of the soaps impregnated with chemical disinfectants are very much more costly than plain soaps, though as disinfectants they are of no greater value.—*Therapeutic Gazette.*

